# Practical considerations to establish a validated platform for pooled detection of SARS-CoV-2 by droplet digital PCR

**DOI:** 10.1371/journal.pone.0271860

**Published:** 2022-11-04

**Authors:** Sofía Heckel, Antonella Pacini, Franco Paredes, Ma. Victoria Petreli, Marilina Perez, Natalia Adriani, Guadalupe Ibarra, Hugo Menzella, Alejandro Colaneri, Juliana Sesma

**Affiliations:** 1 Molecular Biology Department, Hospital Provincial de Rosario (HPR), Rosario, Santa Fe, Argentina; 2 Instituto de Inmunología Clínica y Experimental de Rosario (IDICER-CONICET), Rosario, Santa Fe, Argentina; 3 Facultad de Ciencias Bioquímicas y Farmacéuticas de Rosario (FBioyF), Rosario, Santa Fe, Argentina; 4 Instituto de Procesos Biotecnológicos y Químicos Rosario (IPROByQ), Rosario, Santa Fe, Argentina; 5 Consejo Nacional de Investigaciones Científicas y Técnicas (CONICET), Buenos Aires, Argentina; 6 Facultad de Ciencias Médicas (FCM-UNR), Rosario, Santa Fe, Argentina; Imam Abdulrahman Bin Faisal University, SAUDI ARABIA

## Abstract

Detection of SARS-CoV-2 has created an enormous workload for laboratories worldwide resulting in a restriction at the time of massive testing. Pool testing is a strategy that reduces time and costs. However, beyond the detection of infectious diseases in blood banks, this approach is rarely implemented in routine laboratories. Therefore, what was learned from the SARS-CoV-2 pool testing should represent an opportunity to increase diagnostic capabilities. The present work, carried out in the context of a diagnostic laboratory of a public hospital during the COVID-19 pandemic, represents a contribution to this end. The main limitation of pool testing is the risk of false negatives that could have been identified by individual tests. These limitations are the dilution of samples with a low virus load during pooling and that the integrity of the sample may be affected by the quality of the sample collection. Fortunately, both limitations coincide with the main strengths of droplet digital PCR (ddPCR). ddPCR is a third-generation PCR that splits the amplification into thousands of droplets that work in parallel, increasing sensitivity and resistance to inhibitors. Therefore, ddPCR is particularly useful for pool testing. Here we show how to factor between test sensitivity and savings in test time and resources. We have identified and optimized critical parameters for pool testing. The present study, which analyzed 1000 nasopharyngeal samples, showed that the pool testing could detect even a single positive sample with a CT value of up to 30 in pools of 34 samples. This test was performed using three different standard extraction methods, the simplest being heating only, which resulted in substantial savings of extraction reagents in addition to PCR reagents. Moreover, we show that pooling can be extended to use saliva, which is less invasive and allows self-collection, reducing the risk for health personnel.

## Introduction

The updated analysis shows that the COVID-19 pandemic has caused more than 6 million deaths to-date reported to the World Health Organization (WHO) becoming a great threat to public health worldwide [1, 2]. Moreover, the International Monetary Fund projected a global cumulative output loss of nearly $13.8 trillion through 2024 [3, 4]. Today, it continues to be a hazard as variants keep emerging with increased transmissibility and severity, reduced effectiveness of treatments and partial resistance to vaccines [5, 6]. Therefore, the implementation of national plans for the detection of positive cases was necessary to guarantee timely care for the patients and stop further spread of the virus. The high percentage of asymptomatic patients complicates the situation since it is not possible to base the diagnosis solely on the clinic, requiring a laboratory diagnosis [7].

Vaccination was shown to reduce the viral load and the spread of the disease, but even when vaccination campaigns are showing excellent results [8], a close follow-up to check for their efficacy is needed [2]. Thus, scaling up and maintaining large testing capacities worldwide remains a challenge, due to high costs, limited reagents, and scarcity of test equipment and laboratory staff [9].

Real Time quantitative polymerase chain reaction q-PCR (RT-qPCR) is the gold standard method for infectious disease detection like COVID-19 as it is a highly specific and sensitive diagnostic [10]. One possibility to increase the number of tests to detect SARS-CoV-2 is to adopt the pool testing strategy developed by Dorfman [11, 12]. Pool testing is a method of grouping several samples to be analyzed in a single reaction test. Practical experience shows that countries that carried out massive testing campaigns were the most effective in controlling the pandemic [13]. Therefore, pool testing has become an appealing method that has been explored by many laboratories using RT-qPCR [13–24].

Even when it possesses several advantages (it is cost-effective at low prevalence of the virus [15] and expands screening capacities [21]), pool testing by RT-qPCR also presents limitations like low sensitivity due to dilution effect [15] and inhibitions [16] associated to this method. Both limitations could be circumvented by a third-generation PCR: the droplet digital PCR (ddPCR). In this end-point PCR, the sample is divided into thousands of nanodrops, amplified, counted by a droplet reader and the result is analyzed by a Poisson distribution. Therefore, quantification by ddPCR is absolute, more resistant to inhibitors and more sensitive than RT-qPCR, favoring its use for pool testing [1]. Although ddPCR doubles the costs of RT-qPCR, the use of pooling reduces the cost when three or more samples are pooled, and then the fall of the operational costs has an inverse correlation with the number of samples mixed in the pools [25]. An additional advantage of ddPCR is that since worldwide SARS-CoV-2 detection is mainly done by RT-qPCR and resources are often lacking, ddPCR makes available a different diagnostic approach.

There are many different strategies to calculate the optimum size of the pool for a given prevalence [26–28]. In general, the lower the prevalence, the bigger the size, as long as the sensitivity of the method allows it. Therefore, at a low level of prevalence, ddPCR pool testing would be useful as an epidemiological surveillance system for the early detection and isolation of confined outbreaks caused by new SARS-CoV-2 variants, and recursively tracking each contact to control a possible epidemic focus.

The information provided in this study may serve other laboratories to establish a platform for the detection of SARS-CoV-2 in pooled samples of nasopharyngeal swabs and saliva by droplet digital PCR as a cost-effective method [29].

## Materials and methods

### Sample collection

Both Nasopharyngeal and saliva samples were collected between 1^st^ April and 31^st^ July 2021. Nasopharyngeal swabs were collected by healthcare providers, mixed with 2 mL saline solution, and sent to the Hospital Provincial de Rosario’s molecular biology laboratory. For the saliva samples, we followed a protocol designed previously in our facilities. Briefly, saliva was collected after 3 hours of fasting by passive drooling. All samples were stored at 4°C until extraction. In Biosafety Level 2 containment, RNA was inactivated and then extracted using the Applied Biosystems™ MagMAX™ Viral/Pathogen II (MVP II) Nucleic Acid Isolation Kit (Cat# A48383) and the KingFisher™ Flex Purification System, SmartStart, 96 deep-well head (Cat#5400610). When validating the robustness of the method, two other extraction methods were used in parallel: manual extraction with Tiagen® columns (Cat#DP315-R) and an in-house heat-developed method (10 min at 95°C).

### Individual RT-qPCR test

RT-qPCR was performed in the clinical laboratory to detect the presence of SARS-CoV-2 RNA with the PerkinElmer SARS-CoV-2 RT-qPCR Reagent kit® (Cat # 3515–0010), following manufacturer instructions. We received deidentified positive and negative samples with a code. The RT-qPCR step was critical as it was used to prepare negative pools. These pools were tested by ddPCR to assure their negativity. Samples with CT 30 (nasopharyngeal swabs) and CT 33 (saliva) were used as positive samples to spike the negative pools. The size of the pools varied depending on the experiment.

### Pooling preparation

Two different protocols were applied to determine which was the most sensitive pool preparation. For pooling after RNA extraction, RNA extraction was done individually with the Thermo Scientific™ KingFisher™ purification system prior sample grouping. Negative samples were pooled and aliquoted and a single positive sample was added. Pools were analyzed by ddPCR. For pooling before extraction, 1 single positive sample (nasopharyngeal swab in saline solution) was pooled with negative samples before automatic RNA extraction. Saliva samples were pooled after RNA extraction.

### Pooled ddPCR test

All procedures followed the manufacturer’s instructions for the Bio-Rad SARS-CoV-2 ddPCR Kit (Cat#12013743) unless otherwise specified. In a 96-well PCR plate, 5,5 μL of the extracted patient sample (representing 200 μL of the original sample), pool sample, or control were added to 16,5 μL of master mix for each reaction. Droplet generation was done in the QX200 AUTO DG (Cat#12001630) following the manufacturer’s instructions. Then, the samples were placed on a Bio-Rad TX100 Touch Thermal Cycler for reverse transcription and PCR amplification. The plate was read on a Bio-Rad QX200 Droplet Reader and analyzed using the QuantaSoft™ Analysis Pro 1.0.596 software.

Primers and probes for the N1 and N2 gene targets were present, along with ribonuclease P protein subunit p30 (RNAse P) as an internal control for endogenous human RNA. The N1 probe was labeled with carboxyfluorescein (FAM), the N2 probe was labeled with HEX and FAM, and the RNAse P probe was labeled with HEX fluorophore. Each case was reviewed for automated droplet count and then reviewed manually to determine whether positive droplets fell outside gating parameters. As specified in results, samples that had 3 or more copies/20μL for both N1 and N2 genes were considered positive. Besides the cut-off determined and according to manufacturer instructions, a minimum number of accepted droplets is required to ensure optimal detection in the analysis: positive assays must have at least 6,000 accepted droplets, negative assays must have 10,000 or more, and samples with fewer than 10 positive droplets for RNAse P gene are considered invalid. If this is not accomplished, then the test must be repeated.

### Statistical analysis

Analysis of the ddPCR data was performed with QuantaSoft analysis software v.1.7.4.0917 (Bio-Rad) to calculate the concentration of the targets. Regarding statistical analysis, to assess significant differences Wilcoxon and Friedman’s tests were performed with GraphPad Prism 8.00. A p-value <0.05 was considered statistically significant.

### Ethical approval

The study protocol was approved on December 10^th^, 2020 by the Bioethics Committee of Universidad Nacional de Rosario, Facultad de Ciencias Médicas (FCM) (resolution: 3733/2022). Signed informed consent was not necessary as deidentified samples of SARS-CoV-2 tests by Rosario Provincial Hospital were used. This study was conducted in accordance with the principles of the 1964 Declaration of Helsinki.

## Results

### Specificity

According to European pharmacopeia [29], specificity is the ability to unequivocally evaluate nucleic acid in the presence of components expected to be present in the sample. To determine the specificity, first, we needed to define our cut-off limit (background). This number represents the positive drops that could appear unspecifically in a negative sample. In order to define our cut-off, we measured 50 samples that contain only the RNAse P gene. Thus, the cut-off limit was determined as the maximum number of positive molecules/20μL (which in this case correspond to copies for the N1 and N2 genes/reaction). Table 1 shows that when the 50 reactions containing only the RNAse P gene were dosed, they presented less than 3 copies/20μL for the N1 and N2 sequences of the N gene. This cut-off limit was used to analyze the specificity of the assay.

**Table 1:** Summary of cut off- and specificity determination.

Assessment		N gene	Total assays per assessment	Number of assays that meet the tested criteria			
				0 copy/20μL for both genes	<1 copy/20μL for both genes	<2 copies/20μL for both genes	<3 copies/20μL for both genes
**Cut- off**	**RNAse P**	**N1**	**50**	**40**	**40**	**48**	**50**
		**N2**	**50**	**39**	**39**	**48**	**50**
**Specificity**	**Negative pool**	**N1**	**100**	**92**	**92**	**100**	**100**
		**N2**	**100**	**84**	**84**	**98**	**100**

Initially, a positive control of RNAse P RNA was used to set up the cut-off limit. As there was certainty that no SARS-CoV-2 genome was present in these samples, any molecule detected by the system must have been nonspecific, thus the cut-off chosen was the one that determined all 50 assays as negatives. With the cut-off assessment, it was possible to differentiate positive samples (those with 3 or more copies/20μL for the N1 and N2 sequences of the N gene) from negative ones. Then, this cut-off limit was used to determine the specificity by testing 100 negative pools. The specificity was 100%.

To test the specificity, 100 negative pools were prepared using negative samples previously determined by RT-qPCR. These negative pools were tested by ddPCR and the cut-off limit was used to define positive and negative pools. When this assay was executed for the first time some drawbacks appeared regarding our system. As defined by Alteri *et al*. [30]: “ddPCR-based assay can allow an efficient detection of SARS-CoV-2 at low copy number in symptomatic cases resulting negative by standard RT-qPCR.” When our assumed negative pools were tested with ddPCR, some resulted in positive pools. These pools were opened and tested individually by ddPCR. There was at least one positive sample in each of these positive pools. Ideally, we should have used samples from patients from before the pandemic to prepare the negative pools but that was not possible as our laboratory emerged due to the pandemic. Then, new negative pools were prepared after individual ddPCR analysis. The assay was repeated using the cut-off previously set, (less than 3 copies/20μL for both sequences N1 and N2), and specificity resulted in 100%. Results are shown in Table 1.

### Maximum pool size

The maximum pool size refers to the higher number of negative samples that can be grouped with a positive sample in a pool and still detected the positive sample in 95% of the tests. It was important to select a positive sample with a clinically relevant CT. Bullard *et al*. observed that the infectious virus was detected only on samples with a high concentration of viral RNA (RT-qPCR positive at cycle threshold (CT) < 24) [31] and reported only 12% residual infectivity after CT 30. Furthermore, in van Kampel work, CT values were converted to Log_10_ RNA copies/mL [32], founding that the probability of isolating infectious virus was less than 5% when viral RNA load was below 4x10^6^ RNA copies/mL. This cut-off was similar to the cut-off of 3x10^6^ RNA copies/mL reported by Wolfel *et al*. [33]. In our study, we decided to use as positive sample the ones with the highest CT that is considered clinically relevant. Therefore, we made the correlation between the CT and copy number obtained by RT-qPCR and ddPCR, respectively and decided to use as a positive sample the ones with CT = 30 as this CT corresponds to 4x10^4^ RNA copies/mL.

Originally, three different size pools were made with samples previously tested: 1/10 (1 positive and 9 negatives), 1/24 (1 positive and 23 negatives) and 1/32 (1 positive and 31 negatives). Six different pools of each size were prepared and tested by ddPCR. Table 2 shows that even in the bigger size pool (1/32), the positive sample (CT = 30) was still detected. Thus, the size of the pools was increased to 1/34 (1 positive and 33 negatives) and 1/36 (1 positive and 35 negatives). For these new size pools, 24 repetitions of each one was performed. Table 2 shows that the 1/34 pool was detected in 100% of the cases, while the percentage of positivity for the 1/36 pool was less than 95%. Therefore, the maximum number of samples per pool was defined as 34.

**Table 2:** Maximum grouping determination.

Pool dilutions	D	ND	Repetitions	Positivity
**1/10**	**6**	**0**	**6**	**100%**
**1/24**	**6**	**0**	**6**	**100%**
**1/32**	**6**	**0**	**6**	**100%**
**1/34**	**24**	**0**	**24**	**100%**
**1/36**	**20**	**4**	**24**	**83%**

Positive samples are consistently detected when diluted with up to 33 negative samples. D: detectable. ND: not detectable.

### Robustness of the method

Robustness is the ability to withstand small but deliberate variations in method parameters. To determine that, we evaluated the following variations: pooling order, different extraction methods and cross-contamination.

#### Pooling order assessment

**Fig 1:**
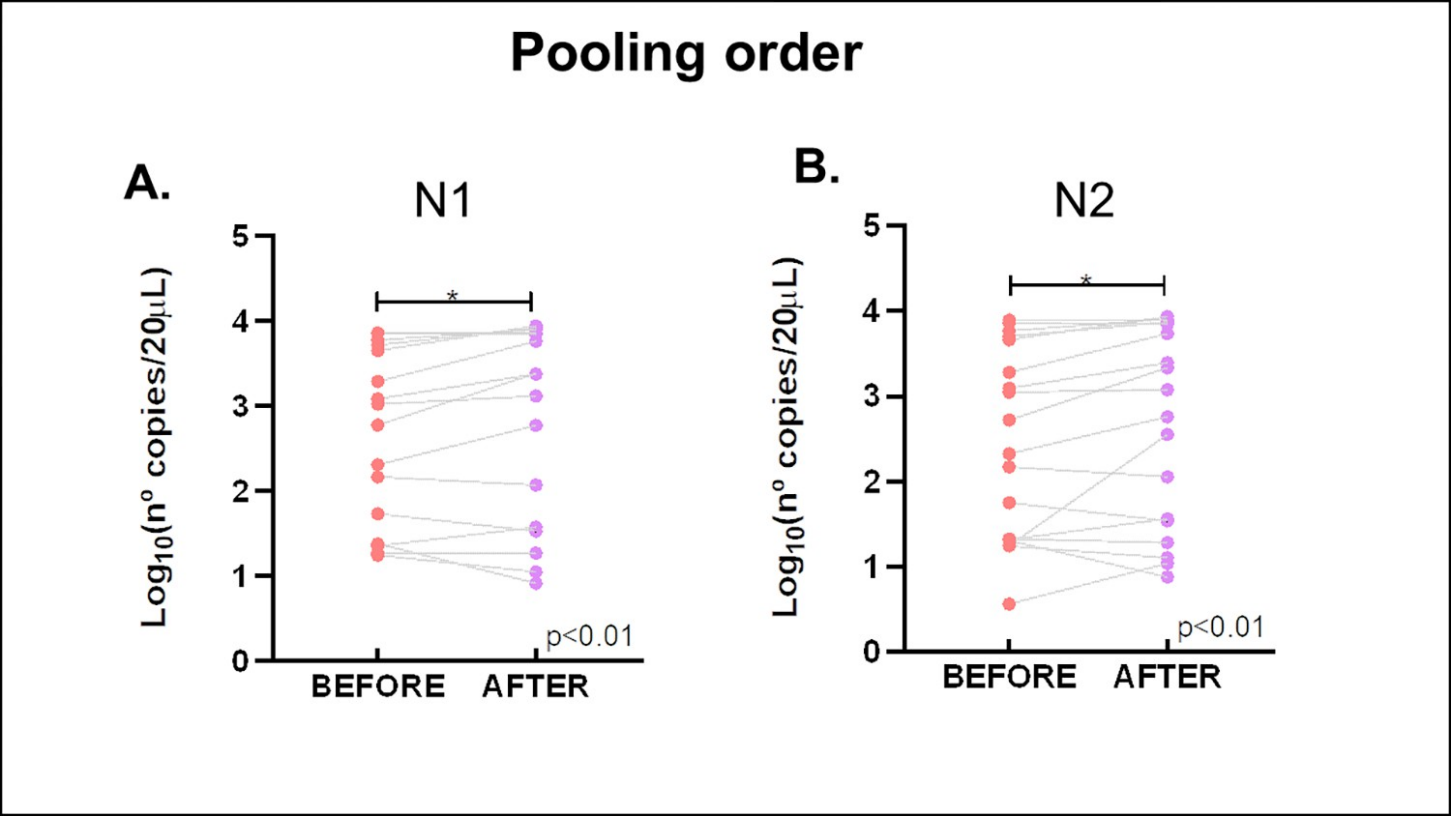
Pooling order assay. N° copies/20 μL were obtained by pooling before or after extraction for A) N1 and B) N2 sequences of the N gene of SARS-CoV-2. The Wilcoxon test and Friedman’s tests for paired, non-parametric samples determined that there is a significant difference (p< 0.01) between both pooling ways.

To prepare the pools before RNA extraction is a considerable reduction of time and work. Therefore, it was important to determine if the sensibility of the pool strategy was still good enough if the pool was made before RNA extraction. To this assay, pool size was 34 and the CT of the positive sample was 30 as discussed before. Pool samples were made prior to RNA extraction and after RNA extraction. To test whether the pooling approach can be applied even before RNA extraction, the nasopharyngeal swab in saline solution of one single positive sample was pooled with the saline solution of 33 negative simples. After that, RNA extraction was performed. In parallel, each sample was purified alone with the same extraction method and then the pool was made. Although, statistical analysis exhibited a significant difference showing greater sensitivity if the pool is assembled after extraction (Fig 1), it was possible to visualize viral RNA in pools prepared both before and after RNA extraction. The concordance between both protocols was 100%. (Shown in Table 3)

**Table 3:** Concordance in pooling order.

Concordance of results in pooling order			
	BEFORE	AFTER	CONCORDANCE RATE
**D**	**20**	**20**	**100%**
**ND**	**0**	**0**	**100%**

Positive samples are consistently detected when diluted with up to 33 negative samples by pooling before or after RNA extraction. D: detectable. ND: no detectable.

#### Different extraction method assessment

Twenty samples were selected with a CT in a range of 20 to 26, greater concentrations than the positive sample used to determine the maximum grouping. Three different extraction methods were tested: manual by column (Tiagen®), automated by magnetic beads (Applied Biosystems™ MagMAX™ Viral/Pathogen II (MVP II) Nucleic Acid Isolation Kit and Thermo Scientific™ KingFisher™ purification system) and heat (10 min. at 95°C).

**Fig 2:**
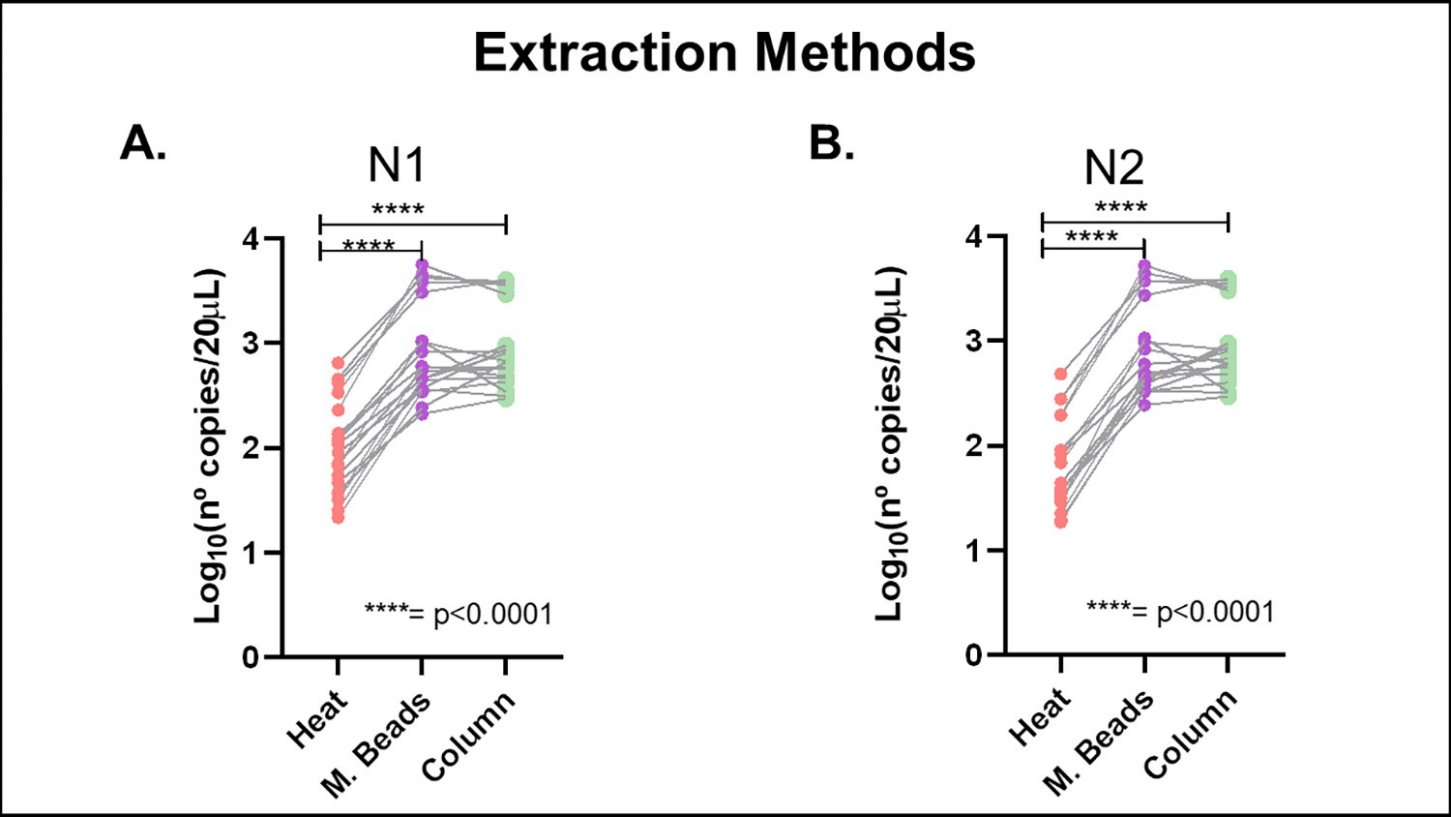
Different methods of extraction assay. N° copies/20 μL obtained with different methods of extraction (heat, manual by column and automated method by magnetic beads) for A) N1 and B) N2 sequences of the N gene of SARS-CoV-2. The Wilcoxon test and Friedman’s tests for paired, non-parametric samples determined that there is significant difference (p< 0.0001) between heat extraction method and the other two methods.

Fig 2 shows that manual extraction by column and automated extraction by magnetic beads present the lower CT values, but even though the CTs varied, viral RNA was detected in all the pools.

#### Cross contamination assessment

To assess the possibility of cross-contamination throughout the process, 20 negative pools were seeded alternated in a plate with 20 pools spiked with a positive sample. Considering the European pharmacopeia [29] specifications, this positive sample had to be present at a viral load concentration 2 order of magnitude higher than the concentration used for the maximum pool. Therefore, since CT = 30 is the highest CT considered clinically relevant, we spiked with a 100-fold more concentrated sample (CT = 23 as 2^7^ = 128), for the cross contamination assessment. Since no positive results were observed in the negative pools when twenty positive pools and twenty negative pools were alternated on the plate, we confirm that there was no cross-contamination (Table 4).

**Table 4:** Seeding method in PCR plate and results.

Copies N°/20μL(N1/N2)						
		1	2	3	4	5
**Pools -**	**A**	**0/0**	**0/0**	**0/0**	**0/0**	**0/0**
**Pools +**	**B**	**45012/46509**	**49147/50151**	**51190/52015**	**55134/53921**	**51779/52955**
**Pools -**	**C**	**0/0**	**3.31/0**	**0/0**	**0/0**	**0/0**
**Pools +**	**D**	**44791/45920**	**43087/44224**	**44879/45943**	**48571/48683**	**46925/47583**
**Pools -**	**E**	**0/0**	**1.56/0**	**1.53/0**	**0/0**	**0/0**
**Pools +**	**F**	**45746/45384**	**53428/55410**	**49796/51500**	**55495/55269**	**43735/44060**
**Pools -**	**G**	**1.59/0**	**0/0**	**0/0**	**0/0**	**2.69/0**
**Pools +**	**H**	**48546/49054**	**55252/56986**	**49552/50586**	**58014/58859**	**51975/52569**

The disposition is related to seeding order on the PCR plate: White and grey cells correspond to negative and positive samples respectively. Each cell shows number of copies/20μL obtained for N1 and N2 genes (expressed as N1/N2).

### Saliva assays

The use of saliva as a sample has several advantages compared to nasopharyngeal swabs. First, there is no need of a trained professional to obtain the sample, reducing the risk of health personnel infection. Second, it is less invasive than swabs and third, there is no need of flocked swabs and transport media which are prone to shortages. Therefore, we analyze the performance of saliva samples arranged in pools. As it is described that saliva has higher content of inhibitors than nasopharyngeal swabs, we started with pools of smaller sizes. We prepared pools of three different sizes: 1/10 (1 positive and 9 negatives), 1/15 (1 positive and 14 negatives), and 1/20 (1 positive and 19 negatives). We decided to use as a positive saliva sample one with CT = 33 (it corresponds to a CT = 30 for a nasopharyngeal sample, as seen in our experiments [34] and considering similarities obtained in bibliography [35]). It was observed that even in the biggest pool prepared (1/20), the positive sample was detected, thus 24 repetitions of 1/20 pools were performed (in different days), and 1/20 was defined as our maximum pool size for saliva with 100% specificity. (Table 5). The decision we made about not increasing the number of samples in the pools and keeping the pool of up to 20 samples was due to the fact that we were at the limit of detection (LOD) of the method; we made this inference based on the number of copies for N1 and N2 genes obtained in the 1/20 pools.

**Table 5:** Maximum grouping determination for saliva samples.

Pool dilutions	D	ND	Repetitions	Positivity
**1/10**	**3**	**0**	**3**	**100.00%**
**1/15**	**3**	**0**	**3**	**100.00%**
**1/20**	**27**	**0**	**27**	**100.00%**

Positive saliva samples are consistently detected when diluted with up to 19 negative samples. D: detectable. ND: no detectable.

### Use of the validated method

During the months of September-October 2021, when positivity rates decreased to 3% in Argentina, this new methodology was implemented in the laboratory. We prepared pools with regular samples that arrived at the hospital and the assays were performed following a Matrix-based non-hierarchical pool testing as shown in the example of Table 6. This strategy uses a combinatorial pool-testing approach [36] where a double-entry chart is created and crossmatch information between lines and columns is used to get the exact sample that is positive. Later, the cross-matched samples are run individually by RT-qPCR to re-confirm their positivity. It is important to consider that in the case of having more than an only pair of positive pools, not all crossmatch are going to be positive samples, that is the reason why it is important to have low infectivity rates to make good use of this method. At that time this new methodology helped to reduce time and costs in our laboratory.

**Table 6:** Double-entry chart with analyzed samples (example of a real case).

	Pool 1	Pool 2	Pool 3	Pool 4	Pool 5	Pool 6	Pool 7	Pool 8	Pool 9	Pool 10
**Pool A**	**123–6**	**140–6**	**417–6**	**507–6**	**508–6**	**153–6**	**163–6**	**042–7**	**064–7**	**096–7**
**Pool B**	**178–14**	**401–14**	**410–14**	**591–14**	**510–15**	**511–15**	**512–15**	**135–14**	**169–19**	**344–14**
**Pool C**	**502–14**	**503–14**	**520–14**	**576–14**	**587–14**	**588–14**	**590–14**	**161–12**	**162–12**	**163–12**
**Pool D**	**170–14**	**501–12**	**587–12**	**589–12**	**509–13**	**510–13**	**502–13**	**503–13**	**100–12**	**111–13**
**Pool E**	**136–13**	**355–13**	**392–13**	**81–20**	**396–13**	**150–12**	**397–13**	**398–13**	**402–13**	**403–13**
**Pool F**	**404–13**	**103–20**	**426–20**	**501–13**	**504–13**	**507–13**	**508–13**	**590–12**	**175–13**	**183–13**
**Pool G**	**101–11**	**102–11**	**111–11**	**028–12**	**503–12**	**505–12**	**087–12**	**102–12**	**107–12**	**504–12**
**Pool H**	**508–12**	**159–5**	**405–6**	**410–12**	**411–6**	**412–6**	**530–6**	**551–6**	**558–6**	**559–6**

Samples that were used for the preparation of 18 pools are shown in this table (numbers in the cells are internal codes of identification of patients used in the hospital). Pools 1–10 were prepared with samples described under each column and pools A-H were prepared with samples described following each line. Considering this distribution and preparation of pools, each sample was tested twice, once in pool 1–10 and once in pool A-H. In grey we show the “putative positive samples” to be retested by RT-qPCR after finding that pools 1,2,6, A, B and E were positives by ddPCR.

In the example shown here, after analyzing by ddPCR the pools described in Table 6, we were able to determine 6 positive pools: 1, 2, 6, A, B and E. The intersection between columns and files shown in grey in Table 6 are the “putative positive samples” to be re-tested by RT-qPCR assays.

Table 7 shows the results obtained after RT-qPCR assay. It was possible to identify three positive samples without further inconvenience. It is important to remark the reduction of time and cost of this validation, considering that from an initial amount of 80 samples, only one ddPCR assay with 18 pools and one RT-qPCR assay with 9 individual samples were done. In other words, only 26 determinations for 80 samples.

**Table 7:** RT-qPCR assay (example of a real case).

Sample	N-gene	ORF1ab-gene	Internal Control	RESULT
	(CT)	(CT)	(CT)	
**123–6**			**36.29**	**ND**
**153–6**			**30.31**	**ND**
**178–14**	**31.12**	**30.20**	**31.28**	**D**
**511–14**			**33.32**	**ND**
**136–13**			**27.54**	**ND**
**150–12**	**30.92**	**29.51**	**31.85**	**D**
**140–6**	**17.18**	**15.73**	**30.27**	**D**
**401–14**			**32.87**	**ND**
**355–13**			**33.80**	**ND**

Samples that were suspected of being positive after pool analysis by ddPCR, were retested individually by RT-qPCR. CT results for N gene (detected with FAM fluorophore), ORF1ab gene (HEX) and RNAse P (Cy5) are shown for each sample, and the resolution after evaluating CT information, is determined in the last column, being D: detectable. ND: no detectable.

## Discussion

This clinical validation of ddPCR for SARS-CoV-2 molecular testing in pool samples demonstrates the feasibility of detecting one positive nasopharyngeal swab sample with a CT = 30 in a pool of 33 negative samples with 100% sensitivity and 100% specificity. According to our experiments, this is the maximum grouping possible that maintains this level of sensitivity. Idan Yelin *et al*. [14] evaluated multi-sample pools with RT-qPCR and determined that viral RNA can be consistently detected but pooling would reach a sensitivity of 96% for a pool size of 16 samples and higher-fold pooling have an estimated false-negative rate of 10%. This is consistent with results from Tao Suo *et al*. [1] who described that ddPCR for SARS-CoV-2 detection is around 500 times more sensitive than RT-qPCR at low-level viral load. This advantage of ddPCR is derived from its use of endpoint quantification rather than quantification by real-time amplification used by RT-qPCR. Moreover, this qualitative measurement for each droplet reduces the susceptibility to poor amplification efficiency, and the compartmentalization of the individual droplets helps to get higher confidence at low input viral load [37]. It is important to highlight the difference between the limit of detection of ddPCR and RT-qPCR. According to one of the studies of Tombuloglu et al. [38], the RT-qPCR method for the diagnosis of SARS-CoV-2 has a LOD of 0.81 copies/μL for the N gene and 1.40 copies/μL for the RdRP gene. In another study [39], Tombuloglu et al. determined that the LOD for RdRP and E genes were at least 101 and 103 copy/μL, respectively by RT-qPCR. On the other hand, Jing Xu’s group [40] determined that the LOD for SARS-CoV-2 by ddPCR was 0,05 copies/ μL for N1 and N2 gene. Thus, even if considering the lowest LOD mentioned for RT-qPCR, ddPCR is still 16 times more sensitive.

Furthermore, ddPCR compared with RT-qPCR, has increased resistance to inhibitors due to lower competition/inhibition for reaction resources derived from the compartmentalization. In nasopharyngeal swabs samples, this technical advantage was proven useful as it was possible to detect a positive sample in a 1/34 pool prepared with crude lysate from patient samples (Heat 10 min. at 95°C). This was evaluated before [37] and adds information in highlighting the potential advantages of ddPCR for COVID-19 diagnosis, particularly to obtain quantitative results from crude lysate, thus obviating the need for nucleic acid purification.

To take full advantage of inhibitor resistance, we show that pure saliva samples can be analyzed by ddPCR, whereas this is not possible by RT-qPCR due to its high inhibitor content. Working with saliva presents several advantages, permits more affordable and frequent testing, fewer risks of infection to healthcare personnel compared with swab-based methods, and alleviates demand for supplies of swabs and personal protective equipment (PPE). For all this, we consider the successful detection of SARS-CoV-2 in saliva in pools of 20 samples, as a significant result of maximum value [41].

Moreover, the process of sample partitioning has the extra advantage of concentrating rare species present in bulk of large quantity of wild type as it increases the relative concentration in the micro-environment of each droplet (each PCR vessel only contains an average of less than 1 molecule) [2]. In our laboratory, ddPCR is being validated to detect SARS-CoV-2 variants (delta and omicron) in a pool of positive samples in order to provide epidemiologically significant results.

When selecting the CT value for the positive sample, we decided to choose the "worst-case scenario". Therefore, our goal was to use the highest CT value that is clinically relevant. Anika Singanayagam’s group [42] demonstrated that infectiousness persists for a week or more after symptom onset, declining over time (with CT = 28 the first week after infection, CT = 30.65 during the second week after infection, and CT = 31.60 after 14 days post-infection). They observed that ten days after symptom onset, the probability of culturing the virus declined to 6.0%. Therefore, we decided to take a CT value close to the second week of infection (lower infective-viral load). Similarly, La Scola *et al*. [43], found a significant relationship between CT value and culture positivity rate. The culture positive rate decreased progressively according to CT values to reach 12% at CT = 33. No culture was obtained from samples with CT ≥34. As mentioned in the results, the group of Poliquin observed infectiousness only with CT < 24 [31] while van Kampen *et al*. described that the probability of isolating infectious virus was less than 5% when viral RNA load was below 4x10^6^ RNA copies/mL [32]. Considering all this information; the fact that in our hands CT = 30 corresponds to 4x10^4^ RNA copies/mL, and having in mind that a CT = 30 is the highest CT value accepted for sequencing and variant determinations in Argentina, we used a CT = 30 as the positive sample for the assays. For saliva samples, the CT value chosen was 33, as previous evidence from our lab demonstrated that there is an increase of 3 cycles from nasopharyngeal swabs to saliva [34].

The asymptomatic and pre-symptomatic patients are likely to be a hidden source of infectious virus; thus, pooling is especially useful for routine community surveys and for monitoring cohesive groups. Local and global epidemic response critically depends on determining carriage frequency in the population, which is greatly enabled by pooling techniques [14].

Additional advantages of pooling with ddPCR are the reduction in cost and time. Even when ddPCR doubles the costs of RT-qPCR, these costs are reduced when pooling samples. Savings will depend on the positivity rate, the group size, and the cost of ddPCR testing. Considering 1000 samples in groups of 10, for positivity rates of 1% and assuming ddPCR is 2 times more expensive than individual RT-qPCR test, cost reductions of at least 70% and time savings of at least 75% are achievable when comparing ddPCR versus RT-qPCR. Similarly, for a positivity rate of 0.1%, cost savings would be 80% and saving in time remains the same.

Regardless of all advantages mentioned before, our study has some limitations that need to be considered. First, as mentioned by Juing Xu, false negatives are a concern for pooled specimen testing, but studies have found that most borderline positive results occur in convalescent patients after 10 to 14 days of symptoms when infectiousness is considered to be low [40]. Thus, if our primary goal with group testing is to detect infective patients, then our purpose is accomplished. Second, if we take into consideration pre-analytical variables, with pooling we are dismissing invalid samples. The rate of invalid samples/total samples by RT-qPCR in the routine of our lab is 1.3%, and considering that ddPCR is more resistant to inhibitors, this percentage may be even lower. Nevertheless, this problem arises in every pool testing diagnostic strategy and recommendations are to pay special attention to adequate sample collection so that pre-analytical processes do not lead to major signal losses [36, 44]. Then, if we take into consideration that pooling by ddPCR is thought for screening, this has an even less negative connotation.

To sum up, we have validated the use of pooled testing by combining up to 34 nasopharyngeal swab samples per pool by ddPCR, determining a sensitivity of 100%, a specificity of 100%, and the robustness of the method (tolerate pooling before purification and allows different purification methods). Likewise, the maximum grouping for saliva was 20 samples, detected with 100% specificity and 100% specificity. All in all, we proved that ddPCR is a promising molecular diagnostic tool to detect low levels of SARS-CoV-2 RNA by pool strategy. Our hope is that this will allow for more routine monitoring and detection of low carrier frequency, monitoring critical sectors that must kept functional, such as hospitals, workplaces, schools, and sports teams.
